# Exploring Enhanced Cell-Based Therapy for Ischemic Heart Disease and Heart Failure

**DOI:** 10.3390/jcm11133837

**Published:** 2022-07-01

**Authors:** Rosalinda Madonna

**Affiliations:** Department of Pathology, Cardiology Division, University of Pisa, C/o Ospedale di Cisanello, Via Paradisa 2, 56124 Pisa, Italy; rosalinda.madonna@unipi.it; Tel.: +39-050-315-2683; Fax: +39-050-315-2684

Ischemic heart disease (IHD) and heart failure (HF) remain the leading causes of death worldwide [1], despite the great progress made so far through thrombolytic treatments and percutaneous revascularization. This is partly due to the adult heart’s loss of ability to regenerate its own tissue and the inability to prevent scar formation after necrosis. Therefore, new strategies are needed to repair the broken heart. Several attempts have been made with stem cell transplantation, both in the acute and chronic phases. Although it has been shown that stem cell transplantation as such does not lead to the consistent recovery of heart function or to muscle regeneration, modest benefits have been observed. Research is now exploring the benefits of enhanced cell therapy and the delivery of cell products through an off-the-shelf approach. Together with the regeneration of the heart muscle, the formation of new vessels represents an essential step in the regeneration and repair processes after the ischemic insult. Angiogenesis is normally orchestrated by specific supportive cells residing in the heart, the so-called cardiac stromal cells (CSC). These cells play a critical role in maintaining normal heart function, contribute to cardiac remodeling after an ischemic insult, and orchestrate the heart’s repair processes [2]. CSCs are supportive cells capable of communicating with cardiomyocytes through the release of paracrine factors that regulate cardiac metabolism and angiogenesis and provide cardioprotection [3,4,5]. These cells can be isolated based on the expression of several cell surface markers, including alkaline phosphatase (Alpl), stem cell antigen-1 (Sca1), platelet-derived growth factor receptor beta (PDGFRbeta), and neuronal antigen-glial 2 (NG2) [6]. In particular, the positivity for Alpl, PDGFRbeta, and NG2 7 identifies a subpopulation of CSC, the so-called pericytes, capable of playing a fundamental role in the formation of new vessels [6,7,8,9]. These cells are also identified as mesangioblasts. In preclinical studies on the murine model of acute myocardial infarction after coronary ligation, the injection of mesangioblasts into the left ventricle resulted in a recovery of cardiac function of approximately 50% [8]. Whatever the mechanism that leads to improved heart function, aging and cardiovascular risk factors have a negative impact on the healing potential of cardiac cells [10]. In particular, aging and diabetes determine the senescence of cardiac cells, both cardiomyocytes and CSCs. This reduces or modifies the paracrine activity of CSCs, undermining the ability to cross-talk between CSC and cardiomyocytes, hindering their cardioprotective and reparative functions, and ultimately contributing to heart failure. It therefore appears that patients may potentially benefit from therapies that restore senescence, an approach referred to as “anti-senescence” or “rejuvenating” therapies [11,12]. Our research groups had experience with one such “rejuvenating” approach, the transfection of adipose tissue-derived stromal cells with gene encoding for telomerase (i.e., the catalytic subunit of telomerase reverse transcriptase or TERT) and myocardin (i.e., MYOCD, the nuclear transcription factor for myogenic genes) [3,13,14,15,16]. Our approach is an example of a new strategy that would allow for aged adipose tissue to be replenished by rejuvenating existing cells or by injecting their cell products capable of supplying ischemic tissue with new vessels to prevent ischemic tissue damage [13]. Integrating strategies to either rejuvenate the CSC or regenerate the infarcted heart represent future therapy for IHD and HF.

As a Guest Editor, I thank the authors, reviewers, and the *JCM* team.
